# Supplementary material for: Health behaviors and care seeking practices for childhood diarrhea and pneumonia in a rural district of Pakistan: A qualitative study
Source: PLoS One. 2023 May 16;18(5):e0285868. doi: 10.1371/journal.pone.0285868 (PMC10187914; doi:10.1371/journal.pone.0285868)
Supplement: Attachment — Submitted filename: Response to Reviewers.docx [file pone.0285868.s003.docx]

| **Comments** | **Responses** |
| --- | --- |
| **1**. Please ensure that your manuscript meets PLOS ONE's style requirements, including those for file naming. | We have edited the manuscript according to the template guideline:  <https://journals.plos.org/plosone/s/file?id=wjVg/PLOSOne_formatting_sample_main_body.pdf> |
| **2.** Please include a complete copy of PLOS’ questionnaire on inclusivity in global research in your revised manuscript. Our policy for research in this area aims to improve transparency in the reporting of research performed outside of researchers’ own country or community. The policy applies to researchers who have travelled to a different country to conduct research, research with Indigenous populations or their lands, and research on cultural artefacts. The questionnaire can also be requested at the journal’s discretion for any other submissions, even if these conditions are not met.  Please find more information on the policy and a link to download a blank copy of the questionnaire here: <https://journals.plos.org/plosone/s/best-practices-in-research-reporting>. Please upload a completed version of your questionnaire as Supporting Information when you resubmit your manuscript. | Completed |
| 3. Thank you for stating the following in the Competing Interests section: "The authors declare that the research was conducted in the absence of any commercial or financial relationships that could be construed as a potential conflict of interest."  Please confirm that this does not alter your adherence to all PLOS ONE policies on sharing data and materials, by including the following statement: "This does not alter our adherence to  PLOS ONE policies on sharing data and materials.” (as detailed online in our guide for authors <http://journals.plos.org/plosone/s/competing-interests>).  If there are restrictions on sharing of data and/or materials, please state these. Please note that we cannot proceed with consideration of your article until this information has been declared.  Please include your updated Competing Interests statement in your cover letter; we will change the online submission form on your behalf. | We have added the required statement in our cover letter, and the “Conflict of Interest” section in our manuscript (Page 25):  “The authors declare that the research was conducted in the absence of any commercial or financial relationships that could be construed as a potential conflict of interest. This does not alter our adherence to  PLOS ONE policies on sharing data and materials.” |
| 4. PLOS requires an ORCID iD for the corresponding author in Editorial Manager on papers submitted after December 6th, 2016. Please ensure that you have an ORCID iD and that it is validated in Editorial Manager. To do this, go to ‘Update my Information’ (in the upper left-hand corner of the main menu), and click on the Fetch/Validate link next to the ORCID field. This will take you to the ORCID site and allow you to create a new iD or authenticate a pre-existing iD in Editorial Manager. | Added |
| **Reviewer 2:** | **We would like to truly thank the reviewer for going through the manuscript in such detail, and for all the relevant suggestions which have helped make the methods clear and manuscript strong.** |
| 1. Please avoid using words or statements that blame population or participants throughout the manuscript. E.g. in the absyract, you said, " population fails to recognize...", please rewrite. " | Thank you for bringing this to light, we have rewritten all such lines. |
| 2. Plus, please dont use non-research terms. E.g. ..."ill equipped..."....please write it! | Thank you, we have edited these terms. |
| 3. Separate data collection from analysis. Provide separate and detail report for both methods. The rigor of qualitative studies should be evident from detail reporting. | We have now separated the two sections and added relevant details in both. |
| 4. Sampling of participants is less reported. Please make it detail. | Edited to add more detail (Page 5-6). |
| 5. You mentioned two different method of data analysis: thematic analysis and content anlysis. Which one did you use. Please stick to one. Content anlysis is a design, not data analysis method. Write in detail how you did thematic anlysis. Was it open coding or priori coding approach you used during thematic analysis? How many levels of themes have been emerged from the data? Did you have subthemes or further categories of subthemes? It appears to be one level of theme reported in this study, please add more levels. This will help you to provide thick description for the themes and ensures understanding for the readers. | Thank you for highlighting these important points.  We have now edited this and specified that we used thematic analysis.  We have included two levels of themes:  Themes: 1. Community characteristics and social networks, 2. community mobilization, 3. incentives, 4. childhood diseases, 5. IYCF, 6. immunization, 7. WASH, 8. access to health facilities and providers, and 9. health systems  Subthemes: Diarrhea and pneumonia (for theme#4), access to health facilities, access to health providers, barriers and facilitators (for theme #8), training and capacity building, supply and logistics, HMIS, referral system (for theme#9)  Some topics have subthemes while some do not. We have not added sub-themes where there was general information and creating sub-themes would dilute the core messages of the theme. |
| 6. What do you mean by using two opposing approaches of research design: deductive and inductive by this same qualitative study? Qualitative appears to be inductive in general. So, please elaborate how this study implemented inductive approach. Please remove the idea of deductive. If you mean that you had some eliciting concepts for developinh stidy guides, please say so instead of mentioning deductive. It is not uncommon to have elicting concepts in inductive or qualitative studies. | Yes, the reviewer is very right that we wrote deductive as some themes had been identified as they were present in the study tool (e.g., incentives, community mobilization, childhood diseases). Further themes and subthemes were created as data was coded and analyzed.  We have corrected and detailed the thematic approach in the “data analysis” subsection (Page 7). |
| 7. Dont use " questionnaire" in qualitative studies. Please say study guides or tools. | Edited, thank you. |
| 8. Have a separate subsection for " Rigor" in the methods section. You need to elaborate the crediblity, depedendablity, transferrablity, and confirmablity of the study in that section. The ideas of prolonged engagement, tick deacription, refelexivity, peer debriefying, member checking, subjective neutrality,......many more techniques of qulity assurance throughout the study processes....need to be elaborated. Readers often use these points to judge the quality of your work. | Thank you for your detailed feedback. We have added a “Rigor” subsection (Page 8).  Extended fieldwork/ prolonged engagement  Triangulation of sources  Reliability  Peer review  Low-inference descriptors |
| 9. Please rewrite the manuscript by using COREQ (consolidated reporing systle for qualitative study) guideline. You can find the gudeline in the PLOSEONE or any other journals. | Thank you, we have done this, and attached the COREQ checklist detailing where the required information can be found within the manuscript (non-track changed version). |
| 10. Table 2. Please avoid reporting individuals in the FGD. Use ranges. | In table 1 and line 168, we have clarified the numbers included are for total FGDs and IDIs conducted, not individuals. |
| 11. Please provide subthemes and categories to the presented themes. | The figure shows the broad themes, while the detailed themes and sub-themes are mentioned in the draft. |
| 12. Please add more quotations as you provide subthemes to the themes | We have now added more quotations. |
| 13. What is common and distinct for diarrhoea and pneomonia in this finding? | Added (Page 14). |
| 14. Connected comment 13, how do the distiction inform the interventions differently? | Added (Page 23-24; line 571 – 576). |
| 15. Provide limitation of the study at the end of discussion | Added ‘Limitations’ towards the end of the discussion (Page 25). |
